# Roles of XRCC2, RAD51B and RAD51D in RAD51-Independent SSA Recombination

**DOI:** 10.1371/journal.pgen.1003971

**Published:** 2013-11-21

**Authors:** Heïdi Serra, Olivier Da Ines, Fabienne Degroote, Maria E. Gallego, Charles I. White

**Affiliations:** Génétique, Reproduction et Développement, UMR CNRS 6293 - Clermont Université - INSERM U1103, Aubière, France; Karlsruhe Institute of Technology, Germany

## Abstract

The repair of DNA double-strand breaks by recombination is key to the maintenance of genome integrity in all living organisms. Recombination can however generate mutations and chromosomal rearrangements, making the regulation and the choice of specific pathways of great importance. In addition to end-joining through non-homologous recombination pathways, DNA breaks are repaired by two homology-dependent pathways that can be distinguished by their dependence or not on strand invasion catalysed by the RAD51 recombinase. Working with the plant *Arabidopsis thaliana*, we present here an unexpected role in recombination for the Arabidopsis RAD51 paralogues XRCC2, RAD51B and RAD51D in the RAD51-independent single-strand annealing pathway. The roles of these proteins are seen in spontaneous and in DSB-induced recombination at a tandem direct repeat recombination tester locus, both of which are unaffected by the absence of RAD51. Individual roles of these proteins are suggested by the strikingly different severities of the phenotypes of the individual mutants, with the *xrcc2* mutant being the most affected, and this is confirmed by epistasis analyses using multiple knockouts. Notwithstanding their clearly established importance for RAD51-dependent homologous recombination, XRCC2, RAD51B and RAD51D thus also participate in Single-Strand Annealing recombination.

## Author Summary

The repair of DNA double-strand breaks by recombination is key to the maintenance of genome integrity in all living organisms. Recombination can however generate mutations and chromosomal rearrangements, making the regulation and the choice of specific pathways of great importance. Through modulation of the activity of the recombinase RAD51, the RAD51 paralogue proteins play key roles in the regulation of recombination. Considerable advances have been made in understanding of the RAD51 paralogue proteins and their roles in mediating RAD51-mediated homologous recombination, however very little is known of possible roles that they may have in other recombination pathways. Working with the plant, *Arabidopsis thaliana*, we show here major roles for three RAD51 paralogues in RAD51-independent single-strand annealing recombination. Notwithstanding their clearly established importance for RAD51-dependent homologous recombination, XRCC2, RAD51B and RAD51D thus also participate in Single-Strand Annealing recombination.

## Introduction

DNA double-strand breaks (DSB) are produced by ionizing radiation, free radicals derived from metabolism, DNA crosslinking reagents and during DNA replication [1], [2]. DSB can lead to mutations and rearrangements and/or loss of chromosomes, causing tumorigenesis or cell death. DSB must be repaired to maintain genome integrity, and this is carried out by end-joining through non-homologous recombination or by homologous recombination, which implicates DNA sequence homology of the recombining molecules (for reviews, see [3], [4]). The pathways that utilize homology for repair can be distinguished by their dependence or not on strand-invasion catalysed by the RAD51 recombinase (or DMC1 in meiosis): gene conversion homologous recombination (HR) is RAD51-dependent while single-strand annealing (SSA) is RAD51-independent [3].

RAD51-dependent HR is an error-free DSB repair mechanism involving the use of a homologous template for restoration of the original sequence. It involves resection of the 5′-ended DNA strands at the DSB, generating 3′ single-stranded DNA overhangs that are bound by replication protein A (RPA). Assisted by mediator proteins, RAD51 displaces RPA and forms a helical nucleofilament on the exposed single-stranded DNA (ssDNA) flanking the DSB. This nucleofilament performs the homology search and catalyses invasion of the homologous template DNA, following which the invading 3′ ends are extended through DNA synthesis. The joint recombination intermediate is resolved to separate the recombining DNA molecules and thus restore chromosome integrity (for a review, see [3]).

In addition to RAD51 and the meiosis-specific DMC1, a number of RAD51 paralogue proteins have been described in a variety of organisms. These share 20% to 30% homology with RAD51 and presumably arose by gene duplication and evolved new functions [5]. They clearly play key roles in DNA repair through HR, but their exact functions are not fully understood (for reviews, see [6]–[8]).

Two *S. cerevisiae* RAD51 paralogues, RAD55 and RAD57, form a heterodimeric complex which associates with the RAD51 nucleoprotein filament, stabilising it against disruption by the SRS2 antirecombinase [9]. Recent work has characterized novel yeast RAD51 paralogues: Shu1, Shu2, Csm2 and Psy3, components of the “suppresses *sgs1* hydroxyurea sensitivity” (SHU or PCSS) complex which also promotes RAD51 filament assembly and its stability through counteracting the antirecombination activity of the SRS2 and SGS1 helicases [10]–[17]. Fission yeast has homologues of Shu1, Shu2 and Psy3 (Rlp1, Sws1 and Rdl1) and SWS1 and SWSAP1 are members of a human SHU complex [11], [12], [18].

Five RAD51 paralogues have been identified in animals and plants: RAD51B, RAD51C, RAD51D, XRCC2 and XRCC3 (for reviews, see [7], [19], [20]). Animal cells defective in any of the RAD51 paralogues are hypersensitive to DNA cross-linking agents, such as Cisplatin and Mitomycin C, and show spontaneous chromosomal aberrations [21]–[27]. Mouse *xrcc2*, *rad51b*, *rad51c* and *rad51d* mutants are embryonic lethal [28]–[31]. In contrast, all five RAD51 paralogues Arabidopsis mutants grow and develop normally and *rad51c* and *xrcc3* mutant plants are sterile due to recombination defects [32], [33].

Two-hybrid and co-immunoprecipitation studies have shown that the five RAD51 paralogues form two major complexes: RAD51B-RAD51C-RAD51D-XRCC2 (BCDX2) and RAD51C-XRCC3 (CX3), as well as RAD51B-RAD51C (BC) and RAD51D-XRCC2 (DX2) sub-complexes [5], [7], [8], [34]–[40]. RAD51 paralogue complexes act at both early and late stages of the recombinational repair process, although their exact roles remain to be identified [32], [41]–[48]. The early role of RAD51 paralogues in HR is to promote formation and stabilization of RAD51 nucleoprotein filament (reviewed by [6]–[8], [19]), very probably through counteracting disruption of the filament by helicases [9]–[14]. Recent work shows that the BCDX2 complex, and not the CX3 complex, is responsible for RAD51 recruitment at DNA damage sites in human cells [43]. After RAD51-mediated strand invasion, the RAD51 paralogues influence gene conversion tract length [42], [47] and have been linked to Holliday junction (HJ) resolvase activity [45], [46]. In addition, RAD51 paralogues can bind Y-shaped replication-like intermediates and synthetic HJ, in accordance with a role for RAD51 paralogues in repair during DNA replication and in resolution of HR intermediary structures [49], [50].

The second main pathway using homology for repair, single-strand annealing (SSA), promotes recombination between tandemly repeated DNA sequences flanking a DSB. SSA does not involve DNA-strand invasion and has been shown to be independent of RAD51 [51]–[54]. After bidirectional 5′-3′ resection of the DSB ends, the exposed complementary sequences anneal. Subsequent removal of non-homologous 3′-ended ssDNA tails, filling-in of any single-strand gaps and ligation completes the process. The SSA recombination pathway thus leads to deletion of the interstitial DNA sequence lying between the repeats and one of the repeated homologous sequences (for reviews, see [3], [55]).

Little is known about possible involvement of the RAD51 paralogues in RAD51-independent SSA. Yeast Rad55 and Rad57 are not required for SSA in a plasmid assay [51] or spontaneous direct repeat recombination [56], [57] and a recent study has shown that absence of Rad55, Csm2 or Psy3 result in increased SSA recombination at a direct repeat chromosomal locus in yeast [15]. In Arabidopsis, RAD51, RAD51C and XRCC3 are not required for SSA, although a mild reduction in the efficiency of SSA was reported in the *rad51c* mutant [53].

In this study, we describe an unexpected role in the SSA pathway for Arabidopsis XRCC2, RAD51B and RAD51D, highlighting for the first time a function of these three RAD51 paralogues in RAD51-independent SSA recombination.

## Results

### XRCC2 is required for SSA recombination

Although XRCC2 is known to be involved in RAD51-dependent homologous recombination in both vertebrates and in plants [6], [7], [19], [43], [44], its potential role in RAD51-independent SSA has not been tested.

**Figure 1:**
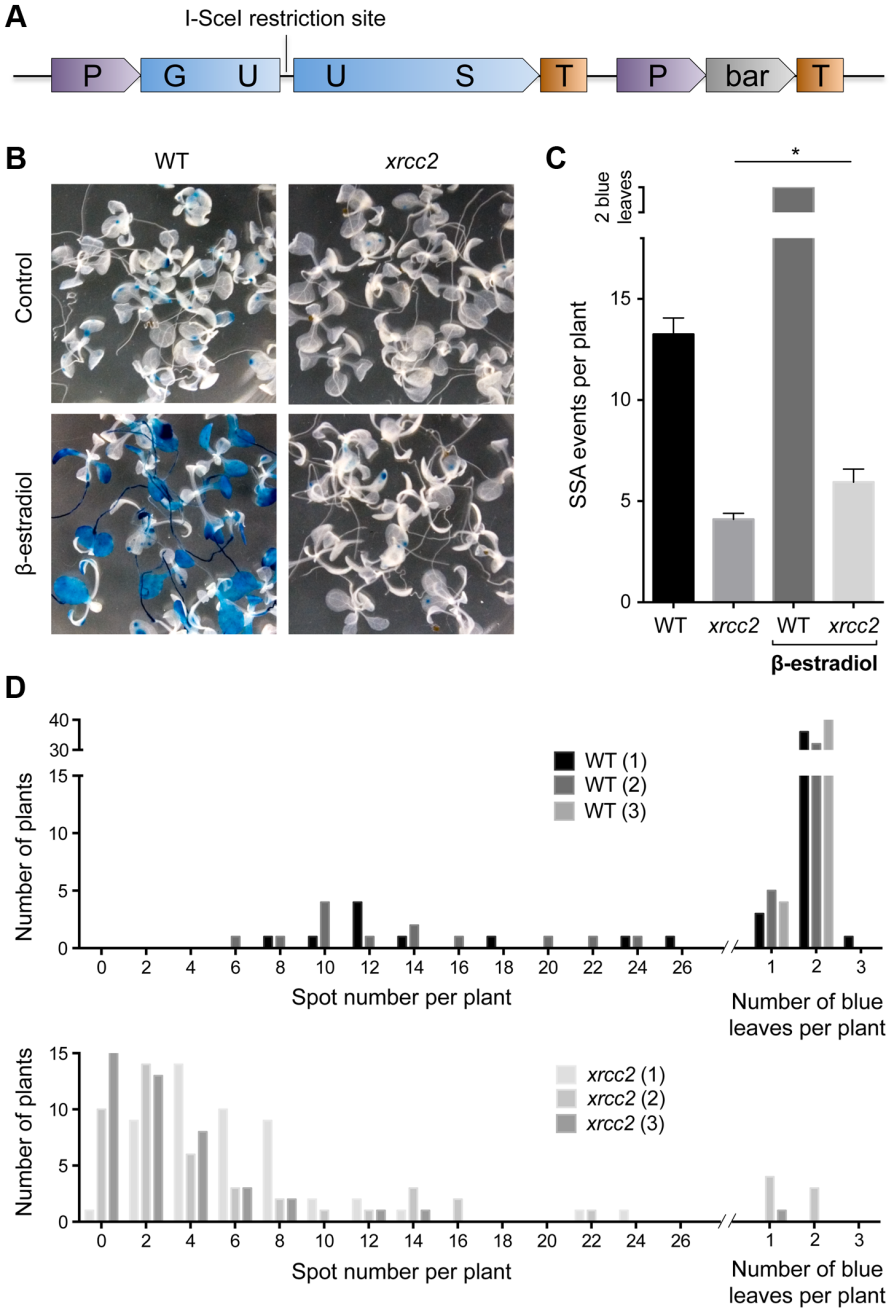
I-SceI induced DGU.US recombination depends upon XRCC2. (A) Schematic map of the recombination substrate DGU.US. (B) β-glucuronidase assay of 14 day-old seedlings grown with or without induction of I-SceI by β-estradiol clearly shows reduced numbers of blue recombinant GUS+ sectors in the *xrcc2* mutant. (C) Quantification of recombination events confirms the role of XRCC2. Bars are mean values ± standard errors. * Significant difference (p = 0.036, Mann-Whitney test). (D) Frequency distributions of recombinant spot numbers per plant of 3 independent WT and *xrcc2* T2 lines grown in the presence of β-estradiol.

SSA recombination was monitored in *xrcc2* mutant *Arabidopsis thaliana* plants using the well-characterised DGU.US recombination reporter locus - consisting of an I-SceI restriction site flanked by 3′ and 5′ truncated copies of the β-glucuronidase gene (GUS) in direct orientation and with an overlap of 557 bp (Figure 1A; [58]). Cleavage of the I-SceI site induces recombination between the flanking GUS sequences and the resulting functional GUS gene is scored histochemically as blue somatic spots. I-SceI induced recombination at this tester locus has been shown not to depend upon RAD51 [53].

We introduced the GUS recombination reporter locus into *xrcc2* mutant and wild-type (WT) plants through crossing and transformed these DGU.US lines with an inducible I-SceI expression cassette (Materials and Methods). Three independent transformants (T2 lines) were selected for each genotype, each with a single insertion site of the I-SceI cassette. Seeds of these lines were plated onto medium containing hygromycin (in order to select plants carrying the I-SceI cassette), in the presence or absence of I-SceI expression inductor (β-estradiol), and numbers of blue GUS+ spots counted after 14 days of growth (Figure 1). Induction of I-SceI expression by β-estradiol treatment in WT plants resulted in a considerable increase of numbers of recombinant blue spots/sectors (Figure 1B and C). In contrast, expression of I-SceI had very little effect on numbers of blue spots in *xrcc2* mutant plants, with means of 5.9 spots per plant in the presence of β-estradiol, and 4.1 in its absence. Repetition of these analyses with two other independent I-SceI transformant lines yielded similar results (Figure 1D). XRCC2 thus clearly plays an important role in the SSA recombination pathway.

**Figure 2:**
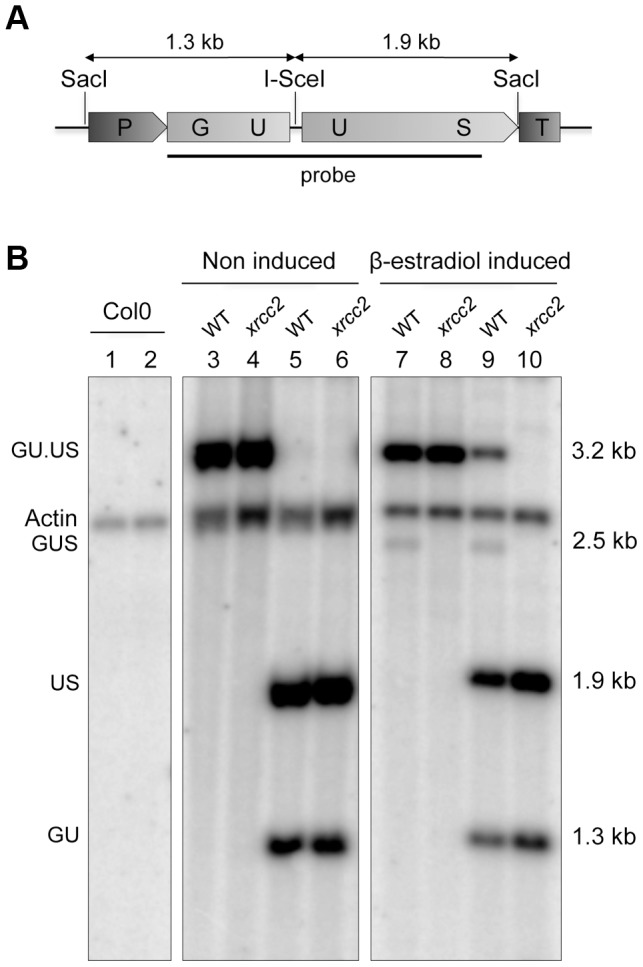
Molecular confirmation of recombination in WT, but not *xrcc2* mutant plants. Schematic representation of the GU.US recombination tester locus (A) and Southern analysis (B) of DNA from plants grown in the absence (lanes 1 to 6) or presence of β-estradiol (lanes 7 to 10), digested with SacI (lanes 1,3,4,7,8) or SacI plus I-SceI (lanes 2,5,6,9,10). The blot was hybridized with a GUS-specific probe as indicated in panel (A). The recombined GUS gene has lost its I-SceI site and is seen as a single 2.5 kb SacI fragment only in DNA from WT plants grown in presence of β-estradiol (lanes 7 and 9). Col0: WT plants of Columbia ecotype.

The histochemical GUS assay is an indirect measure of somatic recombination and we thus carried out Southern analyses to demonstrate directly that the decrease of number of GUS+ spots in *xrcc2* mutant plants is due to a failure of restoration of the GUS gene. Southern analysis was carried out on SacI-digested genomic DNA of WT and *xrcc2* mutant plants (induced or not by β-estradiol). In DGU.US lines, restoration of the GUS gene results in deletion of the repeated sequence, including the inserted I-SceI site. In DNA of WT plants, the reconstituted GUS gene is clearly visible as a band at the expected size (2.5 kb) after induction of I-SceI expression, but not in its absence (Figure 2, lanes 3 and 7). Treatment of the genomic DNA samples with I-SceI *in vitro* prior to electrophoresis confirms that the 2.5 kb fragment has lost its I-SceI site (Figure 2, lane 9), consistent with elimination of the I-SceI restriction site during the restoration of the marker gene. No restored GUS gene was detected in the *xrcc2* mutant line (Figure 2, lanes 8 and 10). This molecular analysis is thus fully consistent with the results of the β-glucuronidase assay and confirms the implication of the XRCC2 protein in the SSA recombination pathway.

We note also the presence of a 3.2 kb band in the SacI+I-SceI digested DNA from WT plants (Figure 2, lane 9). That this I-SceI resistant band is due to *in planta* rejoining of I-SceI breaks through end-joining recombination was verified by PCR amplification and DNA sequencing. Approximately 10% of the sequences carried a mutation at the I-SceI restriction site. DNA sequencing showed that these result mostly from small deletions (Figure S1). As previously described [59]–[63], these events can be ascribed to end-joining exploiting the presence of microhomologies either side of the I-SceI cleavage site.

### XRCC2 function in spontaneous DGU.US recombination does not depend upon RAD51 activity

**Figure 3:**
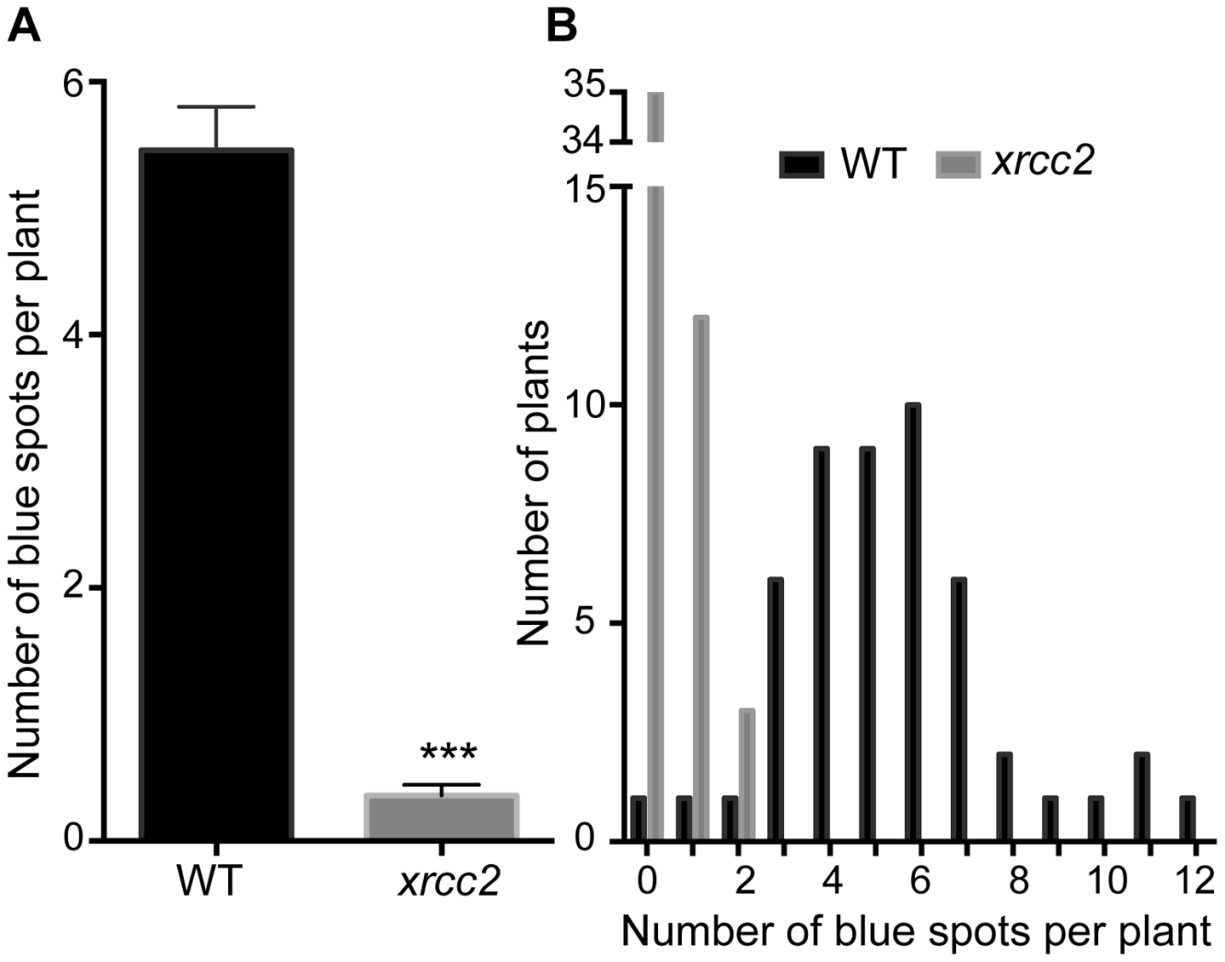
Spontaneous DGU.US recombination is reduced in the *xrcc2* mutant. A significant reduction in spontaneous recombination rate is observed in *xrcc2* mutant compared to WT plants. (A) Mean values ± standard errors of the means. *** p<0.0001 (Mann-Whitney test). (B) Frequency distributions.

Although minor, the DGU.US recombination analyses shown in Figure 1C also showed a difference in numbers of blue spots between WT and *xrcc2* plants in the absence of β-estradiol. To check whether this is due to differences in spontaneous recombination rates or to leakiness of the inducible I-SceI cassette (or both), we monitored recombination in *xrcc2* mutant and WT plants with the same DGU.US locus (at the same location in genome), but which do not carry I-SceI (Figure 3). This analysis showed a reduction of number of recombinant spots in the absence of the I-SceI cassette, for both WT and *xrcc2* plants (from 13.24 to 5.46 and 4.10 to 0.36 spots per plant, respectively; Figures 1C and 3A), confirming the presence of some leakiness in expression of the I-SceI inducible promoter in the absence of β-estradiol. In the absence of the I-SceI cassette, mean numbers of blue spots per plant were however still significantly (15-fold) reduced in *xrcc2* mutants (0.36 ; s.e.m = 0.08) compared with WT controls (5.46 ; s.e.m = 0.34 ; Figure 3). An independent repetition of this experiment confirmed these results (Table 1). XRCC2 is thus clearly involved in spontaneous recombination of the DGU.US substrate.

**Table 1:** Spontaneous DGU.US recombination in *xrcc2* mutant and in wild-type plants.

*Experiment*		*n*	*N*	*m* ± *SEM*	*Ratio xrcc2/WT*
1	WT	50	273	5.46±0.34	
	*xrcc2*	50	18	0.36±0.08	**0.066**
2	WT	50	310	6.20±0.51	
	*xrcc2*	50	19	0.38±0.09	**0.061**

Recombination in the mutants and WT were compared using non-parametric statistical analysis (Mann–Whitney test). Differences between *xrcc2* and WT are highly significant (p<0.0001) in both cases. n, the number of plantlets screened; N, the total number of blue spots (recombination events) ; m ± SEM, the mean number of recombination events per plant ± standard error of the mean.

**Figure 4:**
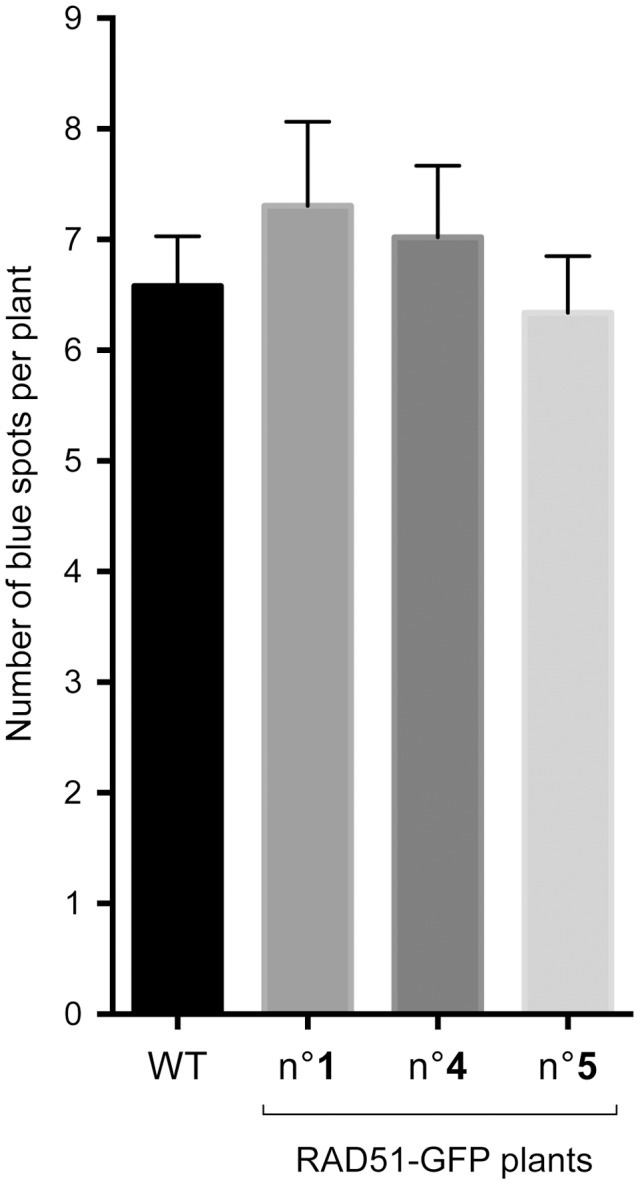
Spontaneous DGU.US recombination is RAD51-independent. No significant effect on spontaneous recombination rate was observed in three independent transformants carrying the dominant-negative RAD51-GFP construct. Bars are mean values ± standard errors.

As mentioned above, I-SceI induced recombination at the DGU.US locus has been shown to be RAD51-independent [53]. This has not however been confirmed for spontaneous recombination, for which different mechanisms can be envisaged - single-strand annealing, intermolecular synthesis-dependent strand annealing, break-induced replication [62], [64], [65]. We thus tested the RAD51-dependence of spontaneous recombination at DGU.US by expressing the dominant-negative RAD51-GFP fusion protein [66]. Plants were transformed with the RAD51-GFP fusion protein construct and three T2 lines each with a single insertion (RAD51-GFP plants) were selected and their *rad51* mutant phenotype tested by verification of sensitivity to the cross-linking agent, Mitomycin C (MMC) (Figure S2). Wild-type plants are not sensitive to the MMC dose used (2% sensitive plants), in contrast to the segregating RAD51-GFP population, in which 76.9% are sensitive. PCR genotyping confirmed that all of the MMC-sensitive and none of the MMC-resistant T2 plants carry RAD51-GFP. Presence of RAD51-GFP is thus perfectly correlated with MMC-sensitivity, confirming the dominant-negative inhibition of RAD51 by the fusion protein [66]. We then tested spontaneous DGU.US recombination in the RAD51-GFP plants (Figure 4). No significant difference was observed in numbers of GUS+ recombinant spots between control and RAD51-GFP plants (Mann-Whitney test) clearly confirming that spontaneous recombination of the DGU.US substrate does not depend upon RAD51 activity.

### XRCC2, RAD51B and RAD51D have non-epistatic functions in the SSA pathway

**Figure 5:**
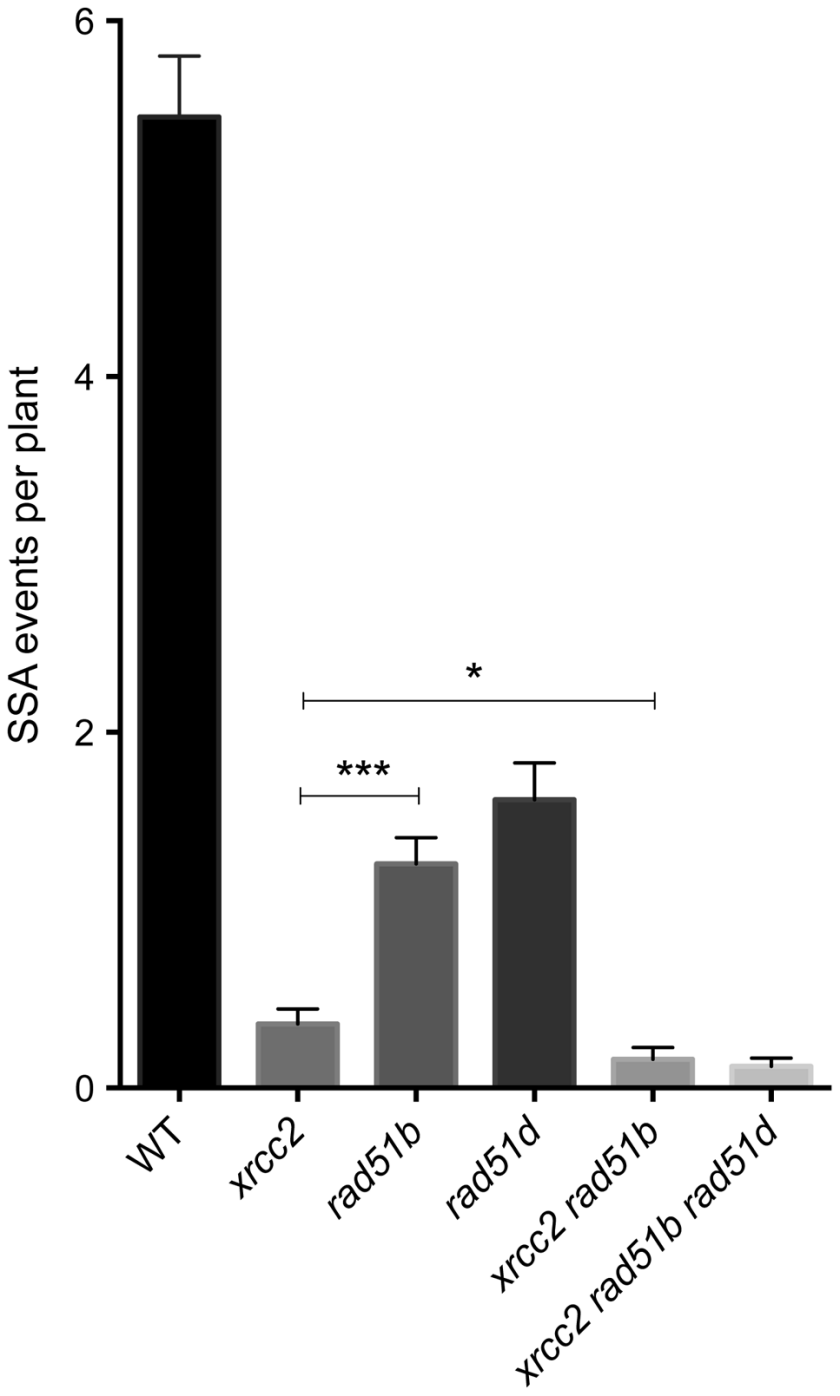
Individual and combined effects of *xrcc2, rad51b* and *rad51d* on spontaneous DGU.US recombination. Significant reductions in spontaneous recombination rate are observed in *xrcc2*, *rad51b* and *rad51d* mutants, and the severities of the reductions differ between these single mutants. A further significant reduction is seen in *xrcc2 rad51b* mutant plants. The triple *xrcc2 rad51b rad51d* mutant shows a further reduction, but this does not differ significantly from that observed in the *xrcc2 rad51b* plants. Bars are mean values ± standard errors. * 0.05<p<0.0001; *** p<0.0001 (Mann-Whitney test).

XRCC2 is one of five RAD51 paralogue proteins, all of which play important roles in recombination [7]. Given the function of XRCC2 in the RAD51-independent SSA pathway presented above, we also tested for evidence of roles of the other RAD51 paralogues, RAD51B and RAD51D, in this pathway. We thus crossed the DGU.US recombination reporter locus into *rad51b* and *rad51d* mutant plants and monitored spontaneous SSA recombination at DGU.US in *rad51b* and *rad51d* mutants. Although less pronounced than the 15-fold reduction observed in *xrcc2* plants, numbers of spontaneous recombination events are also reduced in *rad51b* and *rad51d* mutants (respectively 4.6-fold and 3.4-fold; Figure 5; Table 2) clearly establishing roles for RAD51B and RAD51D in the SSA pathway.

**Table 2:** Spontaneous DGU.US recombination in wild-type, *rad51b*, *rad51d*, double and triple mutants.

*Experiment*		*n*	*N*	*m* ± *SEM*	*Ratio mutant/WT*
1	WT	50	273	5.46±0.34	
	*rad51b*	50	63	1.26±0.15	**0.231**
	*rad51d*	50	81	1.62±0.21	**0.297**
	*xrcc2, rad51b*	50	8	0.16±0.07	**0.023**
	*xrcc2, rad51b, rad51d*	50	6	0.12±0.05	**0.022**
2	WT	50	310	6.20±0.51	
	*rad51b*	50	62	1.24±0.18	**0.200**
	*rad51d*	50	92	1.84±0.23	**0.297**
	*xrcc2, rad51b*	50	6	0.12±0.05	**0.019**
	*xrcc2, rad51b, rad51d*	50	1	0.02±0.02	**0.003**

Recombination in the mutants and WT was compared using non-parametric statistical analysis (Mann–Whitney test). Differences between each mutant and corresponding WT are highly significant (p<0.0001). n, the number of plantlets screened; N, the total number of blue spots (recombination events); m ± SEM, the mean number of recombination events per plant ± standard error of the mean.

Epistasis relationships in SSA recombination between the three RAD51 paralogue genes were tested in *xrcc2 rad51b* double and *xrcc2 rad51b rad51d* triple mutants. Spontaneous SSA recombination was significantly less efficient in *xrcc2 rad51b* double mutants that in the corresponding single mutants (p<0.02) (Figure 5). A slight further reduction in numbers of blue spots per plant was observed in the triple *xrcc2 rad51b rad51d* mutants with respect to the double *xrcc2 rad51b* mutant, but the difference is not significant.

**Figure 6:**
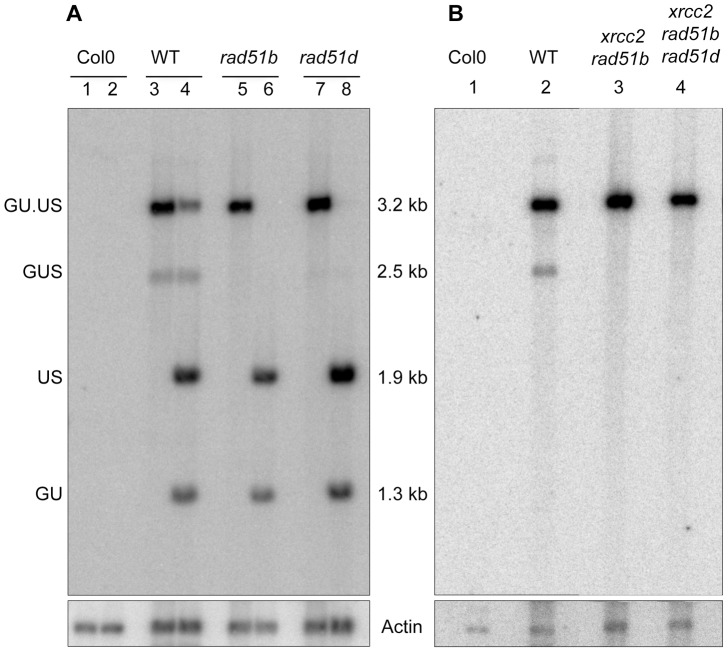
Molecular confirmation of recombination defects in *rad51b*, *rad51d, xrcc2 rad51b* and *xrcc2 rad51b rad51d* mutants. (A) Southern analysis of DNA from *rad51b* and *rad51d* mutant plants grown in the presence of β-estradiol, digested with SacI (lanes 1,3,5,7) or SacI plus I-SceI (lanes 2,4,6,8). (B) Southern analysis of DNA from *xrcc2 rad51b* and *xrcc2 rad51b rad51d* mutant plants grown in the presence of β-estradiol, digested with SacI. The blots were hybridized with a GUS-specific probe. The recombined GUS gene has lost its I-SceI site and is seen as a 2.5 kb band only in DNA from WT plants grown in presence of β-estradiol (A, lanes 3 and 4; B, lane 2). Col0: WT plants of Columbia ecotype.

To confirm at the molecular level the results of the GUS assays, we transformed *rad51b*, *rad51d*, *xrcc2 rad51b* and *xrcc2 rad51b rad51d* mutant plants with the inducible I-SceI expression cassette. Southern analysis of recombination was carried out on β-estradiol induced T2 plants. As expected, the 2.5 kb fragment of the recombination product is only detected in the WT, confirming the GUS assay data (Figure 6).

Arabidopsis XRCC2, RAD51B and RAD51D thus play roles in SSA recombination pathway and these roles are non-epistatic, at least for XRCC2 and RAD51B.

## Discussion

The roles of RAD51 paralogues in RAD51-dependent recombination have been the subject of considerable interest in recent years [6]–[8], [19]. Little is known however of possible roles in RAD51-independent SSA recombination. In Arabidopsis, no effect was found on SSA in *xrcc3* mutants and a barely statistically significant reduction observed in *rad51c* plants [53]. We show here the involvement of three RAD51 paralogues, XRCC2, RAD51B and RAD51D, in RAD51-independent single-strand annealing in *Arabidopsis thaliana*. XRCC2 plays a major role in this pathway with a striking reduction of I-SceI induced recombination and a 15-fold reduction in the number of spontaneous SSA events in its absence (Figures 1 and 3). Spontaneous SSA is also clearly reduced in *rad51b* and *rad51d* mutants (4.6-fold and 3.4-fold reduction respectively; Figure 5; Table 2), although less strongly than in *xrcc2* mutants. The differing severity of the phenotypes of the three mutants is suggestive of individual roles for these proteins, and this is supported by epistasis analyses of double and triple mutant plants (Figure 5, Table 2). An alternative to a direct role of these proteins is that the presence of non-functional RAD51 nucleofilaments in these mutants which might block SSA. The lack of effect on SSA of RAD51-GFP (which forms foci at DSBs and is dominant-negative for GC/SDSA recombination) however argues against this interpretation. Data suggesting differing roles for individual RAD51 paralogues, or sub-complexes, can be found in a number of reports. Individual paralogue mutants in DT-40 cells show non-epistatic phenotypes [67] and biochemical analyses show specific roles for the sub-complexes [68]–[70]. In Arabidopsis, absence of XRCC2 and RAD51B, but not RAD51D, increases rates of meiotic crossing-over [44] and RAD51D appears to be the only RAD51 paralogue to be essential for telomere integrity in human cells [71]. A recent report shows opposing effects on cell-cycle regulation of the inhibition of XRCC3 and RAD51C in HeLa cells, with inhibition of XRCC3 eliciting checkpoint defects and inhibition of RAD51C inducing G2/M cell cycle arrest [48].

What can the roles of XRCC2, RAD51B and RAD51D be in the SSA pathway? The main steps of SSA are (1) bidirectional 5′ to 3′ resection of the DSB ends flanking a DSB, (2) annealing of exposed complementary sequences, (3) excision of non-homologous 3′-ended overhangs, (4) DNA synthesis and (5) ligation which restores two continuous strands [72], [73]. A role in the annealing step is suggested by the capacity of the human BCDX2 complex to catalyse annealing between single-strand DNAs *in vitro* [50]. This study also showed a high affinity of the BCDX2 complex for branched DNA structures, such as Y-shaped DNA, that result from this annealing between tandem repeats during single-strand annealing. Taken together, these results strongly suggest a role of XRCC2, RAD51B and RAD51D in the annealing of the two exposed repeat sequences on either side of the DSB.

Biochemical studies have identified two main complexes of the five RAD51 paralogue proteins in animal and plant cells: RAD51B-RAD51C-RAD51D-XRCC2 and RAD51C-XRCC3 [5], [7], [8], [34]–[40]. No self-assembly of individual RAD51 paralogues have been detected. Analysis of epistasis relationships of RAD51 paralogues in chicken DT-40 cells show that *rad51b* and *rad51d* are epistatic while *xrcc3 rad51d* double mutant cells exhibit an additive sensitivity to ionizing radiation [67], consistent with differential actions of two major complexes in cellular response to DNA damage. That the three RAD51 paralogues involved in SSA are components of the BCDX2 complex suggests this complex is the active species in SSA. However, the differing severity of the phenotypes of the *xrcc2*, *rad51b* and *rad51d* (and *rad51c*; [53]) mutants argues against the implication of the BCDX2 complex as such. The proposed structure of the complex also argues against being the active form in SSA, with protein-protein interaction studies showing that the four proteins are linked in the order: RAD51B-RAD51C-RAD51D-XRCC2 [35]. Absence of RAD51D should thus exclude XRCC2 from the complex, yet SSA in the *xrcc2* mutants is significantly more affected than in *rad51d* (and similarly for *rad51b* versus *rad51c*). This argument also applies to the RAD51B-RAD51C and RAD51D-XRCC2 sub-complexes (for reviews, [7], [8]). Our data thus favour individual roles of XRCC2, RAD51B and RAD51D in single-strand annealing recombination.

The yeast RAD51 paralogues Rad55 and Rad57 are not required in SSA recombination in a plasmid-based assay [51] and a chromosomal assay shows that absence of Csm2, Psy3 (also RAD51 paralogues) or Rad55 favours SSA with respect to gene conversion recombination [15]. The description here of roles for XRCC2, RAD51B and RAD51D in the RAD51-independent SSA pathway thus highlights a difference in the roles of Arabidopsis and yeast RAD51 paralogues in the SSA pathway. Such a difference is also seen in the roles of RAD51 paralogues in meiotic recombination with *psy3* mutants exhibiting a strong hypo-recombination in yeast [12], while absence of XRCC2 or RAD51B increases meiotic crossing-over in Arabidopsis [44].

In conclusion, we describe here an unexpected role in recombination for the Arabidopsis RAD51 paralogues XRCC2, RAD51B and RAD51D. The roles of these proteins are seen in spontaneous and in DSB-induced recombination at a tandem direct repeat recombination tester locus, both of which are unaffected by the absence of RAD51. Notwithstanding their clearly established importance for RAD51-dependent homologous recombination, these proteins thus also participate in RAD51-independent Single-Strand Annealing recombination.

## Materials and Methods

### Plant material

The *Arabidopsis thaliana xrcc2, rad51b* [74] and *rad51d* [44] mutants used in this work have been previously described. A triple *xrcc2/xrcc2 rad51b/rad51b rad51d/rad51d* mutant was crossed with the recombination tester DGU.US-1 line [58] and single, double and triple mutants homozygous for the DGU.US substrate were identified in the F2. Wild-type control plants come from the same crosses.

The I-SceI coding sequence [75] was placed under control of β-estradiol in the plasmid pMDC7 [76] by Gateway cloning. The resulting vector was transferred into *Agrobacterium tumefaciens*, and used to transform the plant lines utilising the floral dip method [77].

### Growth conditions

Surface-sterilized seeds were stratified at 4°C for 2 days and grown *in vitro* on germination medium (0.8% w/v agar, 1% w/v sucrose and half-strength Murashige & Skoog salts (M0255; Duchefa Biochemie, Netherlands)) in a growth cabinet with a 16-h light/8-h dark cycle, at 23°C with 45–60% relative humidity.

The growth medium was supplemented with 170 µM 17-β-estradiol (E2758; Sigma-Aldrich) for induction of I-SceI expression.

### Histochemical staining of GUS expression

Fourteen-day old seedlings grown under standard conditions (supplemented or not with 17-β-estradiol) were harvested and incubated in staining buffer (0.2% Triton X-100, 50 mM sodium phosphate buffer (pH 7.2), 2 mM X-Gluc (5-bromo-4-chloro-3-indolyl-β-D-glucuronic acid; Biosynth), dissolved in N,N-dimethylformamide). Plants were infiltrated under vacuum for 15 min and incubated at 37°C overnight. The staining solution was then replaced with 70% ethanol to remove leaf pigments, and the blue spots were counted under a binocular microscope.

### Mitomycin C treatment

Seeds were sown on plates containing fresh solid germination medium supplemented with 40 µM Mitomycin C (M0503; Sigma-Aldrich). The plates were then incubated for 20 days (23°C, 16-h light). A plant with 3 or more true leaves is considered resistant.

### Plant DNA extraction and Southern analysis

DNA was prepared from seedlings as described previously [78] and 1.5 µg digested with 100 units of SacI or 25 units of I-SceI in a final volume of 200 µl for 15 h. Digested DNA samples were isopropanol precipitated, resuspended in TE, and electrophoresed in 0.8% agarose-TBE gel. Gel was blotted into a positively charged nylon membrane (Hybond-XL, Amersham Biosciences), which was hybridized in 0.5 M phosphate buffer, 7% w/v SDS, 1 mM EDTA (pH 8) and 1% BSA at 65°C. The DNA probes to the GUS gene (a PCR fragment amplified with 5′-TGGATCCCCGGGATCATCTACTTCTG and 5′-AGCCATGCACACTGATACTCTTCACTCC) and the actin gene (a PCR fragment amplified with 5′-GGCTCCTCTTAACCCAAAGG and 5′-TTACCTGCTGGAATGTGCTG) were labelled with [α-^32^P]dCTP using a random priming labelling kit (Megaprime DNA labelling system, Amersham) according to the manufacturer's instructions. Blots were washed with 0.5% SSC, 0.1% SDS solution at 65°C and imaged with a PhosphoImager (Bio-Rad Personal FX).

## Supporting Information

Figure S1 I-SceI induced mutations in end-joining products of DGU.US repair. The unmodified sequence surrounding the I-SceI cut site of the DGU.US recombination tester locus is shown at the top of the alignment, with the I-SceI restriction site boxed and the cut-sites for each strand arrowed. Mutations are highlighted by gray boxes and the size of deletions (bp) is indicated at right. Flanking microhomologies presumably involved in the end-joining of I-SceI induced DSB are underlined.
(PDF)

Figure S2 Sensitivity to Mitomycin C in T2 Rad51-GFP plants. WT and three independent Rad51-GFP T2 transformants were tested for their sensitivity to the cross-linking agent MMC. The dominant-negative effect of the RAD51-GFP allele is clearly visible in the 3∶1 segregating MMC hypersensitivity of the plantlets. (A) photos of the plantlets and (B) quantitation of sensitive versus resistant plants.
(PDF)
