# Supplementary material for: Roles of XRCC2, RAD51B and RAD51D in RAD51-Independent SSA Recombination
Source: PLoS Genet. 2013 Nov 21;9(11):e1003971. doi: 10.1371/journal.pgen.1003971 (PMC3836719; doi:10.1371/journal.pgen.1003971)

Serra_Figure S1

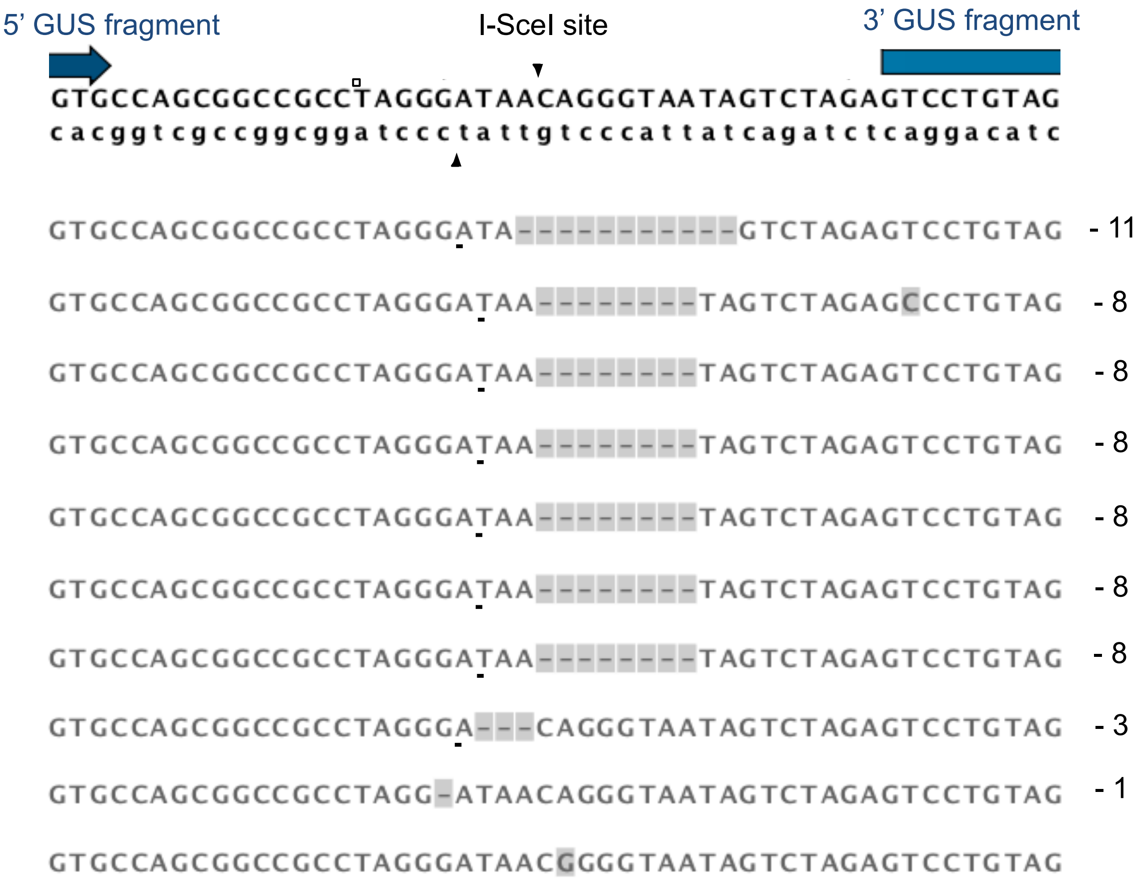

Supplement: Figure S1 — I-SceI induced mutations in end-joining products of DGU.US repair. The unmodified sequence surrounding the I-SceI cut site of the DGU.US recombination tester locus is shown at the top of the alignment, with the I-SceI restriction site boxed and the cut-sites for each strand arrowed. Mutations are highlighted by gray boxes and the size of deletions (bp) is indicated at right. Flanking microhomologies presumably involved in the end-joining of I-SceI induced DSB are underlined. (PDF) [file pgen.1003971.s001.pdf]
