# Supplementary figures and images for: Roles of XRCC2, RAD51B and RAD51D in RAD51-Independent SSA Recombination
Source: PLoS Genet. 2013 Nov 21;9(11):e1003971. doi: 10.1371/journal.pgen.1003971 (PMC3836719; doi:10.1371/journal.pgen.1003971)

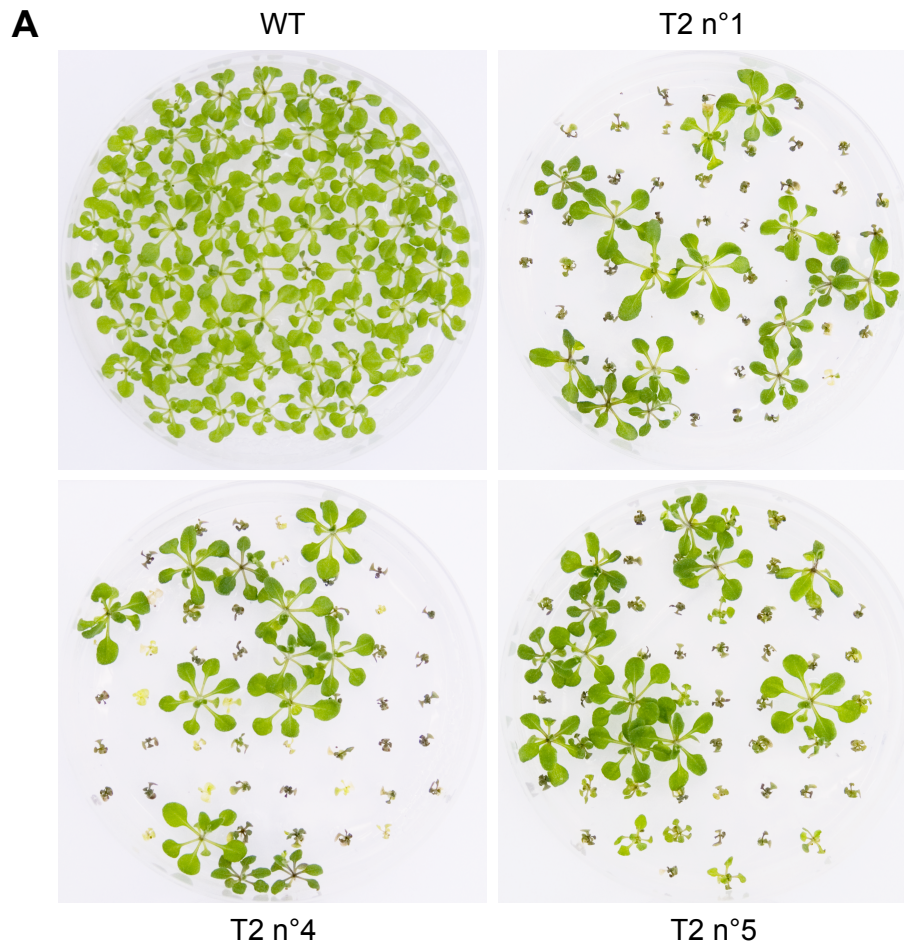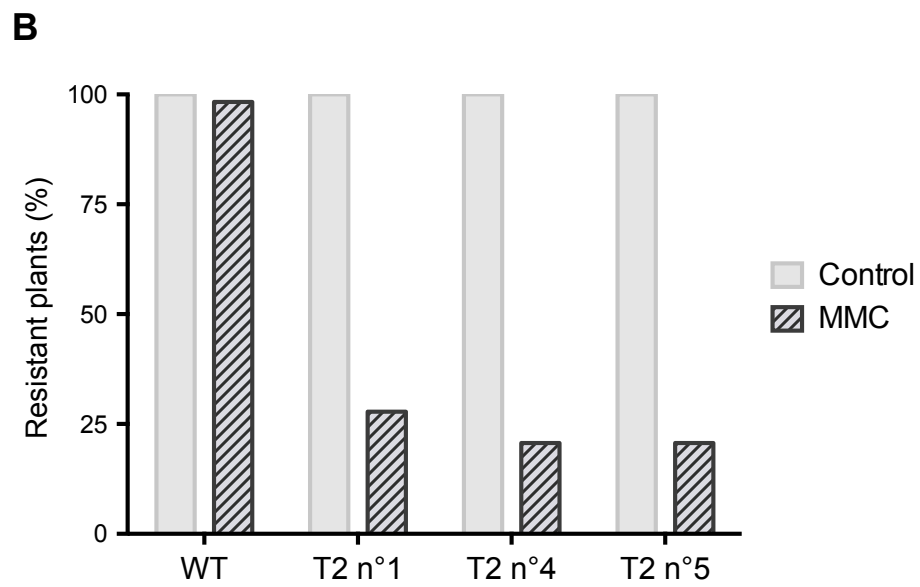

Supplement: Figure S2 — Sensitivity to Mitomycin C in T2 Rad51-GFP plants. WT and three independent Rad51-GFP T2 transformants were tested for their sensitivity to the cross-linking agent MMC. The dominant-negative effect of the RAD51-GFP allele is clearly visible in the 3∶1 segregating MMC hypersensitivity of the plantlets. (A) photos of the plantlets and (B) quantitation of sensitive versus resistant plants. (PDF) [file pgen.1003971.s002.pdf]
